# Selective Chemokine Receptor Usage by Central Nervous System Myeloid Cells in CCR2-Red Fluorescent Protein Knock-In Mice

**DOI:** 10.1371/journal.pone.0013693

**Published:** 2010-10-27

**Authors:** Noah Saederup, Astrid E. Cardona, Kelsey Croft, Makiko Mizutani, Anne C. Cotleur, Chia-Lin Tsou, Richard M. Ransohoff, Israel F. Charo

**Affiliations:** 1 Gladstone Institute of Cardiovascular Disease, San Francisco, California, United States of America; 2 Neuroinflammation Research Center, Department of Neurosciences, Lerner Research Institute, Cleveland Clinic, Cleveland, Ohio, United States of America; 3 Cardiovascular Research Institute, Department of Medicine, University of California San Francisco, San Francisco, California, United States of America; New York University, United States of America

## Abstract

**Background:**

Monocyte subpopulations distinguished by differential expression of chemokine receptors CCR2 and CX3CR1 are difficult to track *in vivo*, partly due to lack of CCR2 reagents.

**Methodology/Principal Findings:**

We created CCR2-red fluorescent protein (RFP) knock-in mice and crossed them with CX3CR1-GFP mice to investigate monocyte subset trafficking. In mice with experimental autoimmune encephalomyelitis, CCR2 was critical for efficient intrathecal accumulation and localization of Ly6C^hi^/CCR2^hi^ monocytes. Surprisingly, neutrophils, not Ly6C^lo^ monocytes, largely replaced Ly6C^hi^ cells in the central nervous system of these mice. CCR2-RFP expression allowed the first unequivocal distinction between infiltrating monocytes/macrophages from resident microglia.

**Conclusion/Significance:**

These results refine the concept of monocyte subsets, provide mechanistic insight about monocyte entry into the central nervous system, and present a novel model for imaging and quantifying inflammatory myeloid populations.

## Introduction

Monocytes, the blood-borne precursors of tissue macrophages and dendritic cells (DCs) [1]–[5], are critical mediators of innate and adaptive immune functions [6], [7]. Monocytes can be divided into at least two subsets based on expression of the surface marker Ly6C in mice [2], [8] and three subsets based on CD14/CD16 expression in humans [9]–[11]. The Ly6C^hi^ [12], [13] and CD14^+^/CD16^−^ subsets [14] express chemokine receptor 2 (CCR2) and low levels of CX3CR1 [2] and appear to be functionally equivalent across species. Conversely, the Ly6C^lo^ and CD14/CD16^+^ subsets lack CCR2 but express high levels of CX3CR1 [2], [3], [14], [15]. Ly6C^hi^ cells enter tissues in response to injury or infection and traffic back to draining lymph nodes [2], [3]. Within inflamed tissues, Ly6C^hi^ cells differentiate into macrophages or conventional DCs [1], [16]. In contrast, Ly6C^lo^ cells patrol the vascular endothelial lumen, respond early to inflammatory insults, and are hypothesized to contribute to wound healing in the later stages of inflammation [1], [16]. CCR2 is thought to mediate the trafficking of Ly6C^hi^ cells [2], [12], [13], [16], [17], while CX3CR1 may contribute to Ly6C^lo^ cell migration [16], although other chemokine receptors such as CCR5 also may be important [18].

The roles of particular monocyte subsets likely vary in different disease models and different organs [19]–[22]. Further complicating the picture, resident macrophages and DCs are difficult to distinguish from macrophages and DCs derived from infiltrating monocytes. The study of monocyte subsets has been greatly aided by the use of CX3CR1-GFP knock-in reporter mice [2], [3], [23], but the lack of reagents to visualize CCR2-expressing cells, particularly in tissue sections, has restricted tracking monocyte subsets in vivo.

To investigate monocyte trafficking in vivo, we generated red fluorescent protein (RFP)-CCR2 knock-in mice, crossed them with CX3CR1-GFP mice, and analyzed them in models of sterile peritonitis and multiple sclerosis using flow cytometry and evaluation of tissue sections. The results provide direct evidence that the presence of CCR2-directed Ly6C^hi^ monocytes dictates the distribution and composition of leukocyte infiltrates in both the brain and spinal cord of mice with experimental autoimmune encephalitis (EAE). The relative expression of CX3CR1-GFP and CCR2-RFP allowed us for the first time to distinguish monocyte/macrophage subsets and discriminate between resident microglia and infiltrating monocyte-derived macrophages in the brain.

## Results

### Generation of *CCR2^RFP^* knock-in mouse

**Figure 1:**
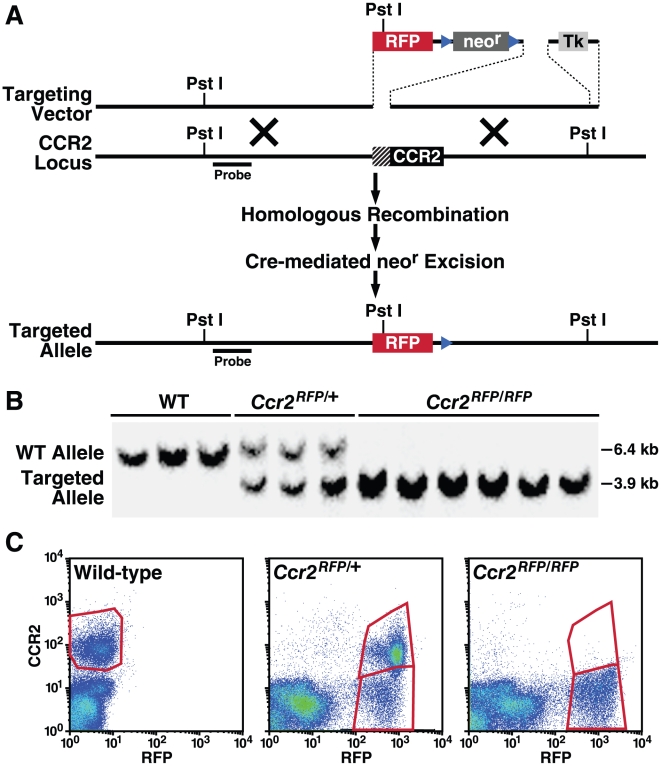
Generation and characterization of CCR2^RFP^ mice. (A) Recombinant targeting strategy. Hatched box represents region of CCR2 deleted by recombination. Triangles represent *loxP* sites. (B) Southern blot analysis of WT, *Ccr2^RFP/+^* heterozygous, and *Ccr2^RFP/RFP^* homozygous genomic DNA. DNA was digested with *Pst*I, and hybridized with the indicated probe. (C) Flow cytometry analysis of peripheral blood leukocytes (PBL) from WT, *Ccr2^RFP/+^*, and *Ccr2^RFP/RFP^* mice. Cells were stained with anti-CCR2 antibody MC21. The results are representative of four similar experiments.

We used an expression cassette with a cDNA that encoded monomeric RFP [24] to replace the first 279 base pairs of the amino terminus of the CCR2 open reading frame (Figure 1A). The CCR2 promoter in the targeting vector was unchanged up to the first methionine of the open reading frame, as determined by sequence analysis. The RFP gene construct included a poly-A termination signal to exclude the remainder of the CCR2 sequence from the mature transcript. Homologous recombinations in embryonic stem cells and mouse derivation were accomplished by standard methods [25], and the neomycin-resistance gene was deleted. Southern blot hybridization confirmed the correct genomic structure (Figure 1B). Flow cytometric analysis of *Ccr2^RFP/+^* and *Ccr2^RFP/RFP^* peripheral blood cells stained for cell-surface CCR2 (Figure 1C) confirmed bi-allelic expression of CCR2 and RFP in heterozygous mice and deletion of CCR2 in *Ccr2^RFP/RFP^* homozygous mice. All CCR2-positive cells were RFP positive, but some RFP-positive cells failed to express CCR2 at the cell surface.

### RFP and CCR2 RNA expression in leukocytes

**Figure 2:**
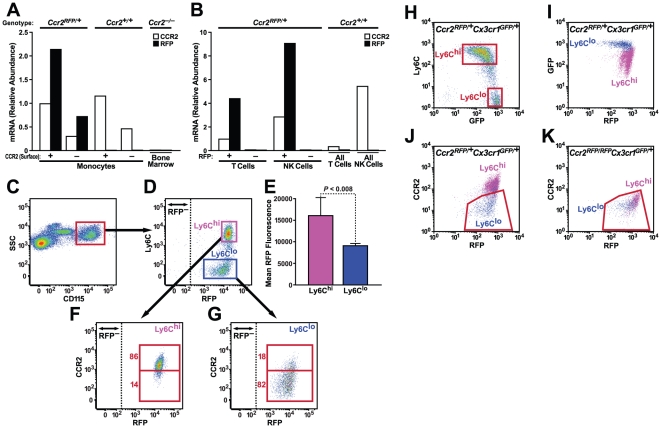
Expression of CCR2 and RFP in sorted cell populations. (A) CD115^+^ monocytes were sorted by flow cytometry into populations that did or did not express cell-surface CCR2. Total RNA was isolated, pooled from three mice per genotype, and analyzed for CCR2 mRNA and RFP mRNA by quantitative RT-PCR. Probe specificity was confirmed with WT and *Ccr2^−/−^* cells. Expression levels were normalized to beta-actin, and the results are expressed relative to the concentration of CCR2 mRNA in monocytes expressing cell-surface CCR2. (B) NK and T cells were sorted by flow cytometry into RFP^+^ and RFP^–^ fractions. Expression of CCR2 and RFP mRNA was characterized as in (A) and normalized to the amount of CCR2 RNA in the RFP^+^ T-cell fraction from *Ccr2^+/RFP^* mice. (C to G) WT, *Ccr2^RFP/+^*, and *Ccr2^RFP/RFP^* mice were bred onto the *Apoe^−/−^* background and fed a high-fat diet for 8 weeks. Peripheral blood monocytes were stained for CD115, CCR2, and Ly6C and analyzed by flow cytometry. (C) Gating on monocytes. (D) Ly6C and RFP expression of monocytes. Dashed line indicates the cutoff for a positive RFP signal. (E) Mean RFP fluorescence intensity in Ly6C^hi^ and Ly6C^lo^ subsets. Bars indicate SEM. n = 4 mice/group. P<0.008. (F) CCR2 and RFP expression of Ly6C^hi^ monocytes. (G) CCR2 and RFP expression in Ly6C^lo^ monocytes. CCR2-positive and CCR2-negative gates were based on CCR2-deficient *Ccr2^RFP/RFP^* mice. (H to K) Flow cytometric analysis of Ly6C, RFP, GFP and cell-surface CCR2 expression on monocytes from *Ccr2^RFP/+^Cx3cr1^GFP/+^* mice. CD115+ monocytes were gated (H) by Ly6C/GFP expression and analyzed for RFP (I) and CCR2 (J) expression. *Ccr2^RFP/RFP^Cx3cr1^GFP/+^* monocytes (K) were used as a negative control for CCR2 surface-staining. Results are representative of four mice in two similar experiments.

CCR2 and RFP transcript levels were determined by quantitative RT-PCR of cell populations isolated by fluorescence-activated cell sorting (FACS). In blood cells from *Ccr2^RFP/+^* mice, CCR2 and RFP mRNA levels tracked closely, as expected for bi-allelic expression from the CCR2 promoter (Figure 2A). RFP transcripts were undetectable in wildtype (WT) cells, and CCR2 was absent in *Ccr2^RFP/RFP^* cells. Monocytes that stained positively for surface CCR2 invariably contained RFP protein and expressed three- to fourfold more CCR2 and RFP transcripts than monocytes not expressing cell-surface CCR2 (Figure 2A). Preliminary flow cytometry surveys of *Ccr2^RFP/+^* mice indicated that some natural killer (NK) and T cells also were positive for RFP (85–95% and 5–10% respectively; data not shown). NK and T cells were sorted into RFP-positive and RFP-negative fractions. Despite lacking CCR2 immunoreactivity by flow cytometry, T cells and NK cells from WT and *Ccr2^RFP/+^* mice contained CCR2 transcripts, and CCR2 and RFP mRNA levels were similar in both cell types (Figure 2B). These results indicate possible post-transcriptional control of CCR2 expression.

**RFP and CCR2 protein expression in monocyte subsets**

To evaluate RFP and CCR2 expression in blood monocyte subsets in an inflammatory setting, we crossed *Ccr2^RFP^* mice with apolipoprotein E–deficient (*Apoe^−/−^*) mice, fed them a high-fat diet for 8 weeks, and analyzed RFP and CCR2 surface expression in CD115^+^ monocytes by flow cytometry (Figure 2C). Both Ly6C^hi^ and Ly6C^lo^ monocytes were RFP-positive, but the Ly6C^hi^ cells were significantly brighter (Figure 2D and E). Approximately 86% of Ly6C^hi^ monocytes expressed cell-surface CCR2 (Figure 2F), as did 18% of Ly6C^lo^ monocytes (Figure 2G). These results indicate that RFP expression can predict CCR2 expression levels in monocytes.

### Cytoplasmic CCR2 in lymphocytes and monocyte subsets

Some CCR2 surface-negative leukocytes contained abundant levels of CCR2 mRNA (and RFP protein and mRNA) (Figure 2). To ask whether Ly6C^lo^ monocytes contained cytoplasmic CCR2 that failed to traffic to the cell surface, we compared CCR2 staining on permeabilized and nonpermeabilized monocytes by FACS (Figure S1). Monocytes from CCR2 knockout mice served as negative controls. In Ly6C^hi^ monocytes, permeabilization increased the CCR2 signal twofold, indicating that approximately 50% of total CCR2 was cytoplasmic, but had little effect on CCR2 staining in Ly6C^lo^ monocytes. Similarly, intracellular CCR2 was undetectable in T cells or NK cells, despite the presence of CCR2 mRNA (data not shown). These results suggest that T cells, NK cells and some Ly6C^lo^ monocytes may be capable of expressing CCR2 protein but do not do so constitutively.

### Analysis of monocytes from *Ccr2^RFP^Cx3cr1^GFP^* mice

To evaluate blood monocyte subsets for expression of CCR2 or CX3CR1 during chronic systemic inflammation, we crossed *Ccr2^RFP/RFP^* mice with *Cx3cr1^GFP/GFP^* mice [23] and examined *Ccr2^RFP/+^Cx3cr1^GFP/+^Apoe^−/−^* mice fed a high-fat diet for 8 weeks. Ly6C^hi^ and Ly6C^lo^ monocytes were both GFP positive (Figure 2H). Ly6C^lo^ cells were clearly brighter than Ly6C^hi^ cells, which had a wider range of GFP intensities. Both Ly6C^hi^ and Ly6C^lo^ monocytes were RFP positive, but Ly6C^hi^ cells were brighter (Figure 2I). There was a broad range of RFP intensities in the Ly6C^lo^ monocytes (Figure 2I), though many were as bright as those in the Ly6C^hi^ population. We next examined RFP expression and CCR2 surface staining on monocytes stratified by their level of Ly6C. As expected from the *Ccr2^RFP^* mice (Figure 2F,G), virtually all of the Ly6C^hi^ monocytes were markedly positive for both RFP and CCR2 staining (Figure 2J). While most but not all Ly6C^lo^ monocytes were negative for CCR2 staining, some Ly6C^lo^ monocytes expressed CCR2 at levels similar to those in Ly6C^hi^ monocytes. *Ccr2^RFP/RFP^* mice served as negative controls to set the FACS gates for CCR2 staining (Figure 2K).

**Figure 3:**
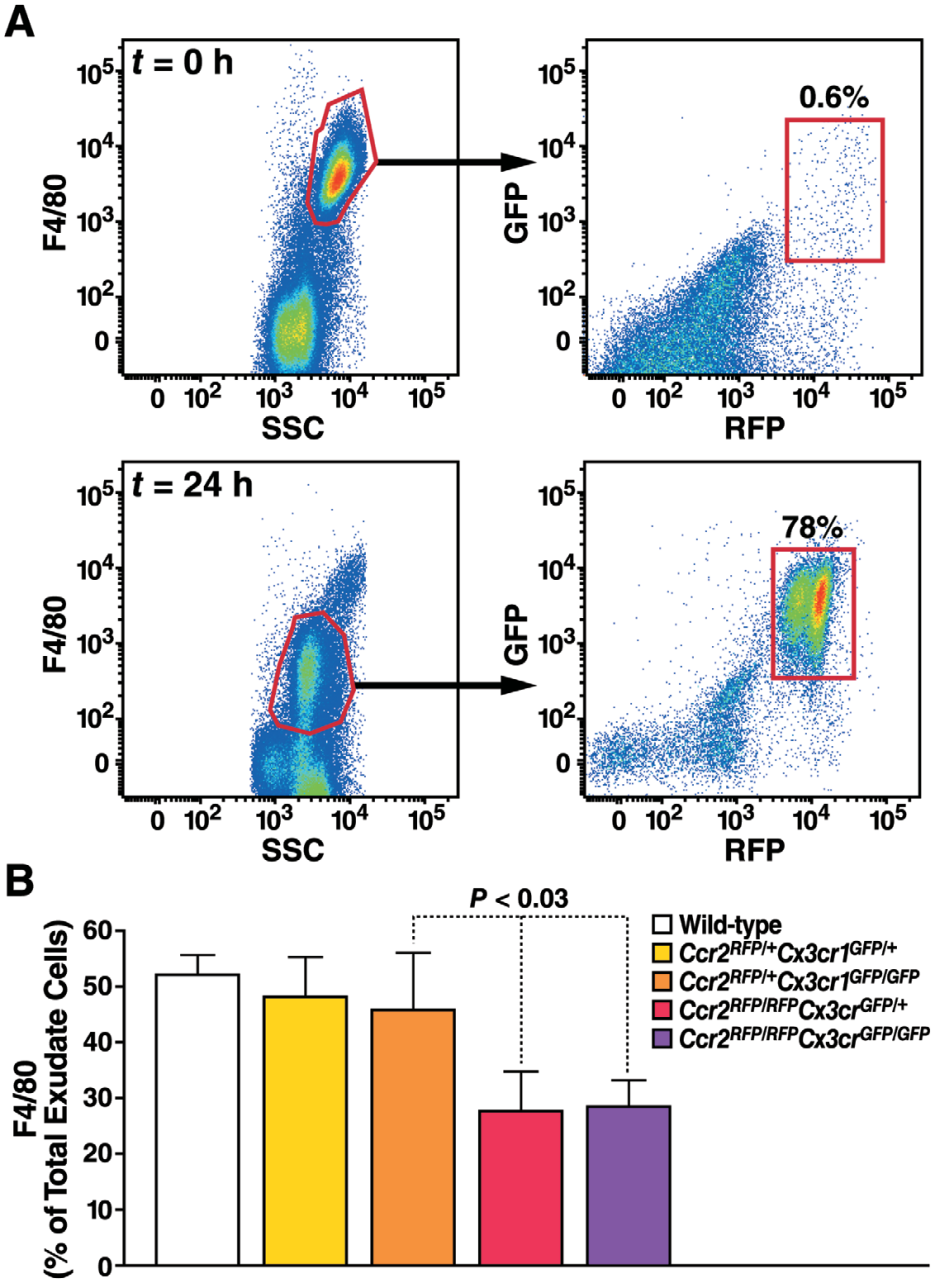
Analysis of peritoneal exudate cells from Ccr2-RFP Cx3cr1-GFP mice in a sterile peritonitis model. WT, *Ccr2^RFP/+^Cx3cr1^GFP/+^*, *Ccr2^RFP/+^Cxc3r1^GFP/GFP^*, Ccr2*^RFP/RFP^Cx3cr1^GFP/+^*, and *Ccr2^RFP/RFP^Cx3cr1^GFP/GFP^* mice were injected intraperitoneally with thioglycollate, and inflammatory exudate cells were harvested 24 h later. Cells were stained with F4/80. (A) RFP/GFP profiles of F4/80^+^ peritoneal cells from naive mice (top panels) and 24 hr after injection (bottom panels). (B) Percentage of exudate cells staining positive for F4/80. Values are mean ± SEM. n = 4 mice/group. Results are representative of two similar experiments.

To investigate the relative contributions of CCR2 and CX3CR1 to monocyte/macrophage recruitment in vivo, we used a mouse model of thioglycollate-induced peritonitis. In naive mice, the vast majority of F4/80^hi^ peritoneal macrophages were negative for GFP and RFP and were probably resident macrophages (Figure 3A). After instillation of thioglycollate for 24 h to induce peritonitis, *Ccr2^RFP/+^Cx3cr1^GFP/+^* F4/80^dim^ monocytes were actively recruited to the peritoneum, and almost all expressed high levels of both RFP and GFP (Figure 3A). Recruited monocytes were Ly6C^hi^ (not shown). In WT and *Ccr2^RFP/+^Cx3cr1^GFP/+^* mice, equal numbers of F4/80^+^ monocyte/macrophages accumulated in the peritoneum (Figure 3B). Monocyte recruitment was little affected by loss of the second CX3CR1 allele *(Ccr2^RFP/+^Cx3cr1^GFP/GFP^*), but was markedly reduced by loss of the second CCR2 allele (*Ccr2^RFP/RFP^Cx3cr1^GFP/+^*) (Figure 3B). Recruitment was indistinguishable in double-knockout mice (*Ccr2^RFP/RFP^Cx3cr1^GFP/GFP^*) and CCR2 knockouts (*Ccr2^RFP/RFP^Cx3cr1^GFP/+^*). Monocyte counts in the blood and bone marrow were also indistinguishable in the two genotypes. These results revealed that the vast majority of monocytes recruited to the peritoneum in response to sterile inflammation are CCR2/CX3CR1 double-positive, although their recruitment is almost exclusively driven by CCR2. These experiments also demonstrated the feasibility of using CCR2-RFP and CX3CR1-GFP to track monocyte subsets in vivo.

### Experimental autoimmune encephalomyelitis in CCR2-deficient mice

**Figure 4:**
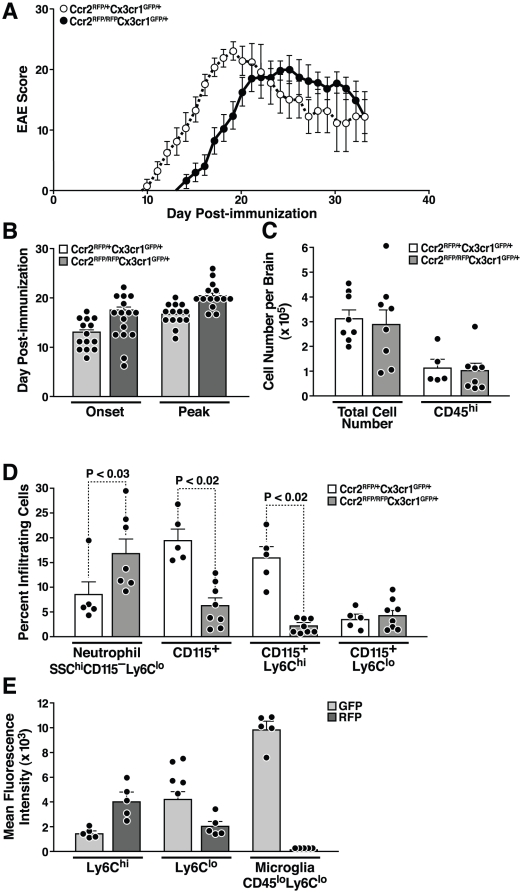
CCR2-deficient mice exhibit delayed onset EAE and decreased monocyte recruitment to the CNS. (A) *Ccr2^RFP/+^Cx3cr1^GFP/+^* and *Ccr2^RFP/RFP^Cx3cr1^GFP/+^* mice were immunized with MOG peptide 33–55 and scored daily for neurological signs. (B) EAE symptom onset and peak disease. (C) Total number of brain mononuclear cells and CD45^hi^ infiltrating cells at peak disease. (D) CD45^hi^ infiltrating cells were analyzed to quantify total neutrophils (SSC^hi^, CD115^−^, Ly6C^lo^), monocytes (CD115^+^), and monocyte subsets (CD115^+^/Ly6C^hi/lo^), (E) CX3CR1 GFP and CCR2-RFP fluorescence intensities in monocyte subsets and microglia. Data points represent individual mice. Values are mean ± SEM. Results are representative of two similar experiments.

To extend these results in an established model of organ-specific inflammation, we compared *Ccr2^RFP/+^Cx3cr1^GFP/+^* and *Ccr2^RFP/RFP^Cx3cr1^GFP/+^* mice with EAE by monitoring disease severity daily for 40 days after immunization. All mice were highly backcrossed onto the C57Bl6 background, and littermates were compared in each experiment. In *Ccr2^RFP/RFP^Cx3cr1^GFP/+^* mice, the onset of EAE was delayed by a mean of 4.5 days (day 17.4±0.6 (SD) vs. day 12.9±0.1 in *Ccr2^RFP/+^Cx3cr1^GFP/+^* mice) (Figure 4A). The maximum disease scores were similarly delayed (day 20.3±0.5 for *Ccr2^RFP/RFP^Cx3cr1^GFP/+^* mice vs. day 16.6±0.4 for *Ccr2^RFP/+^Cx3cr1^GFP/+^* mice), indicating equivalent kinetics of disease evolution in the two groups (Figure 4B). Peak severity scores were lower in the CCR2-null mice, but the difference did not reach statistical significance (p = 0.11). A similar delay in onset followed by equivalent peak severity was observed in *Ccr2^RFP/RFP^Cx3cr1^GFP/GFP^* mice (data not shown). The latter finding indicates that the effect of CCR2 deficiency is dominant on a CX3CR1-null background, given the observation that *Cx3cr1^GFP/GFP^* mice exhibited earlier disease onset than WT mice [26].

### Infiltration by CD115^+^ monocytes is reduced in CNS of CCR2-deficient mice

We previously showed that inflamed EAE tissues contain high levels of CCR2 and CX3CR1 ligands [26], [27], making this tissue ideal for comparing the trafficking properties of monocytes bearing either of the two cognate receptors. To characterize the cellular infiltrate in the CNS of mice with EAE, we used flow cytometry to analyze CNS leukocytes isolated at the peak of disease severity (Figure 4C and 4D). *Ccr2^RFP/+^Cx3cr1^GFP/+^* mice allowed us to simultaneously track CX3CR1-and CCR2-positive cells, and *Ccr2^RFP/RFP^Cx3cr1^GFP/+^* mice allowed us to examine the effect of CCR2 deficiency on monocyte recruitment during EAE. Leukocytes were stained with antibodies against CD45 to distinguish CD45^hi^ infiltrating leukocytes from CD45^lo^ microglial cells. At peak disease severity, similar numbers of total and CD45^hi^ cells were recovered from the brains of each genotype (Figure 4C). CD45^hi^ leukocytes accounted for 36.2±3.5% of total cells in *Ccr2^RFP/+^Cx3cr1^GFP/+^* mice and 35.2±3.5% in *Ccr2^RFP/RFP^Cx3cr1^GFP/+^* mice (*P*>0.5).

To further characterize the inflammatory infiltrate, we stained samples with antibodies against CD115 and Ly6C. Cells were gated by CD45 expression; CD45^hi^ cells were then analyzed for CD115 expression and CD115^+^ cells for Ly6C expression. Among inflammatory CD45^hi^ cells, there were strikingly fewer CD115^+^ monocytes/macrophages in the absence of CCR2 (Figure 4D). In *Ccr2^RFP/+^Cx3cr1^GFP/+^* mice, 88% of monocyte-lineage (CD115^+^) cells were Ly6C^hi^, indicating an overwhelming advantage for this subset in accessing the CNS during EAE (Figure 4D). In *Ccr2^RFP/RFP^Cx3cr1^GFP/+^* mice, however, Ly6C^hi^ cells were drastically reduced, both in number and as a percentage of CD45^hi^ infiltrating cells, demonstrating the importance of CCR2 for recruitment of this subset into the CNS. In contrast, accumulation of the much less numerous Ly6C^lo^cells was unaffected by *CCR2* ablation (Fig 4D). CCR2 deficiency led to an increase in the number of neutrophils, from 8% to 16% of the total infiltrating cells (Figure 4D).

Comparison of CX3CR1-GFP and CCR2-RFP fluorescence intensities in the various CD115^+^ populations from *Ccr2^RFP/+^Cx3cr1^GFP/+^* mice showed that Ly6C^hi^ cells expressed higher levels of CCR2-RFP (*P* = 0.016) than Ly6C^lo^ cells and lower levels of CX3CR1-GFP (*P* = 0.008) (Figure 4E). CD45^lo^ microglial cells were CCR2-RFP negative at EAE peak severity and had the highest level of CX3CR1 expression. Thus, the diversity of the mononuclear myeloid cell infiltrate in EAE is mirrored by a population-specific pattern of CCR2 and CX3CR1 expression, suggesting that the Ly6C^hi^/CCR2^+^ and Ly6C^lo^/CX3CR1^+^ subsets retain functional specialization in the inflamed CNS.

### Differential distribution of CX3CR1 and CCR2 in the naive and inflamed brain

**Figure 5:**
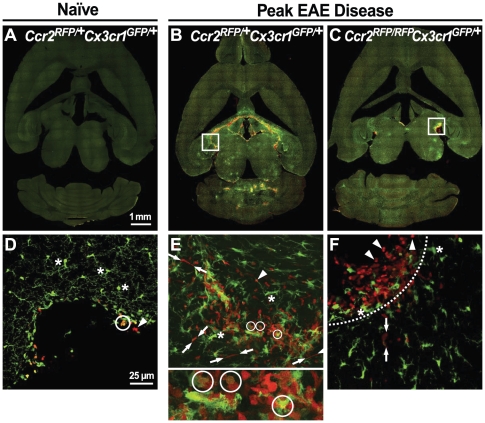
Expression of CX3CR1 and CCR2 in brain lesions of mice with EAE. (A–C) Low-magnification epifluorescence images of naive *Ccr2^RFP/+^Cx3cr1^GFP/+^* (A), diseased *Ccr2^RFP/+^Cx3cr1^GFP/+^* (B), and *Ccr2^RFP/RFP^Cx3cr1^GFP/+^* (C) brains show the extent of inflammation and anatomical location of the lesions. Higher magnification views of the boxed areas are shown in panels D–F. Confocal image of healthy perivascular (D) tissue shows GFP^+^ microglia (asterisks), small round RFP^+^ cells (arrowhead), and double-positive cells (circles). (E) Parenchymal lesion in a *Ccr2^RFP/+^Cx3cr1^GFP/+^*mouse and (F) a perivascular lesion from a *Ccr2^RFP/RFP^Cx3cr1^GFP/+^* mouse at peak EAE disease. Dashed line in (F) indicates the approximate perivascular boundary. Within EAE lesions, note RFP/GFP double-positive cells (circles), GFP^+^ activated microglia (E, F; asterisks), large elongated RFP^+^ cells (E, F; arrows), and small round RFP^+^ cells (E, F; arrowheads). Small RFP^+^ cells were CD3^+^ (not shown). The particularly elongated RFP^+^ cells in the *Ccr2^RFP/+^Cx3cr1^GFP/+^* lesion (E) were much less abundant in the *Ccr2^RFP/RFP^Cx3cr1^GFP/+^* lesion (F).

To visualize CX3CR1-and CCR2-expressing cells in the brains of *Ccr2^RFP/+^Cx3cr1^GFP/+^* and *Ccr2^RFP/RFP^Cx3cr1^GFP/+^* mice, we used fluorescence microscopy. At low magnification, no RFP signal was detected in the brains of healthy *Ccr2^RFP/+^Cx3cr1^GFP/+^* mice (Figure 5A), although a diffuse GFP signal was visible, corresponding to that previously attributed to resident microglia [23]. At high magnification, RFP expression was found in occasional perivascular cells in healthy *Ccr2^RFP/+^Cx3cr1^GFP/+^* brains (Figures 5A and D); flow cytometry, these RFP^+^ cells accounted for <2% of the CD45^+^ marrow-derived cells in naive brain (data not shown). Otherwise RFP expression was absent from the parenchyma of healthy brains (Figure 5D and not shown). RFP^+^ and RFP/GFP double-positive perivascular cells were often in close proximity (Figure 5D) and co-localized in the perivascular zone.

As previously shown by co-localization with the microglial marker iba-1 [28], CX3CR1-GFP was present in microglial cells throughout the brain in both healthy and diseased tissue (Figure 5A–F). In contrast, microglia did not express CCR2 under naive conditions or EAE-associated inflammation. These findings were confirmed by flow cytometry (Figure 4G).

At peak EAE severity, CCR2-RFP^+^ cells were present in the perivascular and parenchymal regions of the brains of *Ccr2^RFP/+^Cx3cr1^GFP/+^* mice (Figure 5B), but were greatly reduced and only found perivascularly in *Ccr2^RFP/RFP^Cx3cr1^GFP/+^* mice (Figure 5C). RFP^+^ cells accumulated in localized lesions, whereas GFP+ cells were both widely distributed and present in clusters within the lesions. Detailed analysis of RFP/GFP expression and cellular morphology in the lesion cores provided further insight into the functions of CCR2 and Ly6C^hi^ monocytes in EAE. The inflammatory lesions contained CX3CR1 single-positive cells with morphological features of activated microglia (larger cell bodies and thicker processes, designated by an “*”, compare Figure 5D and 5E). In *Ccr2^RFP/+^Cx3cr1^GFP/+^* mice, the lesion core contained strikingly elongated CCR2-RFP^+^ cells (arrow, Figure 5E) that were notably less abundant in lesions of CCR2-deficient mice (Figure 5F), strongly suggesting that these cells are derived from Ly6C^hi^ monocytes. A number of CCR2-RFP^+^ cells were also GFP-positive, and thus appear faintly yellow (circled cells, Figure 5E), indicating that both CCR2^+^ and CCR2/CX3CR1 double-positive monocytes invade the brain parenchyma in response to EAE. We noted that the green fluorescence in the monocytes was much less intense than in the microglia, consistent with the flow analysis in Figure 4G. A population of small, round RFP^+^ GFP^−^ cells in EAE lesions of CCR2^+^ and CCR2-deficient mice (arrowheads, Figures 5E–F) was shown in double-labeling experiments to be predominantly CD3^+^ T cells, levels of which were not altered by absence of *CCR2* (data not shown). RFP^+^ cells in the brains of *Ccr2^RFP/RFP^Cx3cr1^GFP/+^* mice were primarily located in the meninges, and RFP^+^ cells were clearly absent from parenchymal tissue (Figure 5F). Microglial cells near *Ccr2^RFP/RFP^Cx3cr1^GFP/+^* lesions displayed a less activated morphology than microglia in *Ccr2^RFP/+^Cx3cr1^GFP/+^* lesions (Figure 5E and 5F), suggesting that monocytic cells in particular contribute to activation of resident microglia.

### Lack of CCR2^RFP^ expression by neuroepithelial cells

The findings of studies using tissue immunohistochemistry and receptor autoradiography have suggested constitutive or inducible expression of CCR2 by neuroepithelial cells (e.g., neurons, astrocytes, neural stem cells) (reviewed in [31]). Aside from sparse perivascular macrophages, we detected no CCR2-RFP^+^ cells in the CNS of healthy mice and no RFP^+^ cells with neuroepithelial morphology in the CNS of *Ccr2^RFP/+^* mice with EAE (Figure 5). Furthermore, CCR2-RFP expression did not overlap with markers of astrocytes, neurons, or oligodendrocytes (data not shown). In analyses of CNS tissues of CCR2-RFP mice, the location and morphology of CCR2-RFP cells provided no evidence of CCR2 expression by CNS neurons. These findings are consistent with recent reports that peripheral nervous system but not CNS neurons inducibly express CCR2 in pain models [32].

### Three color imaging with a myeloid marker

**Figure 6:**
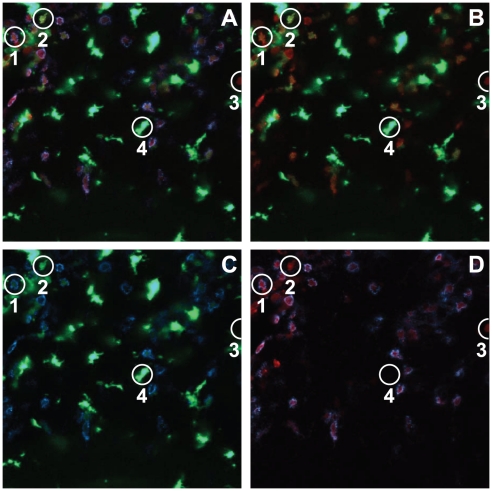
Analysis of monocyte subsets in brain lesions of EAE *Ccr2^RFP/+^Cx3cr1^GFP/+^* mice at peak EAE. Confocal images of the periventricular lesions were obtained after staining with the 7/4 antibody. Images represent the same lesion (A) Merged images of red/CCR2, green/CX3CR1, and blue/7/4. (B) Red/CCR2 and green/CX3CR1. (C) Blue/7/4 and green/CX3CR1. (D) Red/CCR2 and blue/7/4. The majority of CCR2^+^ cells are 7/4^+^ and CX3CR1^lo^ or negative, suggesting that they are classical infiltrating monocytes (see individual cell “1”). Ly6C^lo^ monocytes (CX3CR1^hi^ and CCR2) are also present (cell “2”), as well as T cells (“3”) and activated microglial cells (“4”).

To further analyze the identity of recruited cells, we stained brain sections from *Ccr2^RFP/+^Cx3cr1^GFP/+^* mice at peak EAE for the myeloid marker 7/4. The 7/4 antigen identifies Ly6C^hi^ monocytes and neutrophils, but not NK or T cells. Figure 6A shows a periventricular lesion visualized for CCR2 (RFP, red), CX3CR1 (GFP, green), and 7/4 (Cy5, blue). Figure 6B shows the overlapping CX3CR1-GFP and CCR2-RFP channels only; 6C shows CX3CR1-GFP and 7/4-blue, and 6D shows 7/4-blue and CCR2-RFP, to allow more convenient visualization and comparison of the fluorescent signals. For example, cell “1” in Fig. 6A is 7/4+, RFP^+^ and GFP^dim^, suggesting it is a Ly6C^hi^ monocyte that expresses both CCR2 and CX3CR1. Inspection of Figure 6B confirms that this cell “1” appears yellow (co-localized RFP-GFP), and the green signal is best visualized in Figure 6C. Notably, the intense GFP-green in the microglial cells makes it difficult to visualize GFP-green signal in monocytes, without over-exposing the microglial cells. Inspection of Figure 6D confirms that cell “1”, as well as the vast majority of RFP^+^ cells, are 7/4^+^ and thus likely represents a classical CCR2^+^ inflammatory monocyte. Cell “2” in Figure 6A is 7/4^−^, RFP^+^, and GFP^+^ and is likely a to be a CX3CR1^hi^, CCR2^lo^, Ly6C^lo^ monocyte. Cell “3” is 7/4^−^, RFP^+^, but GFP^−^ and is interpreted as likely to be a T cell. Cell “4” is 7/4^−^, GFP^bright^,and RFP^−^ and has the morphological appearance of a microglial cell.

## Discussion

In this study we created CCR2-RFP knock-in reporter mice and bred them with CX3CR1-GFP mice. Our goals were to quantify CCR2 and CX3CR1 expression in defined monocyte subsets in the blood to visualize infiltrating and resident myeloid cells in naive and challenged mice and to determine how CCR2 contributes to the accumulation of Ly6C^hi^/CCR2^+^ and Ly6C^lo^/CX3CR1^+^ monocytes during autoimmune tissue inflammation in the CNS.

Circulating blood monocytes in the *Ccr2^RFP/+^* mice were uniformly positive for RFP by FACS, and approximately 80% expressed cell-surface CCR2, whereas CCR2 surface-negative monocytes were predominantly Ly6C^lo^, as expected. Mean RFP intensity was approximately twofold higher in Ly6C^hi^ than Ly6C^lo^ monocytes, consistent with reports of relative CCR2 mRNA levels in these populations [3]. The presence of RFP in Ly6C^lo^ monocytes that expressed little or no surface CCR2 protein (>80% of monocytes in this population) raised the possibility that CCR2 was present in the cytoplasm but failed to traffic to the cell surface. However, permeabilization experiments with Ly6C^lo^ monocytes did not reveal substantial intracellular stores of CCR2. Thus, Ly6C^lo^ monocytes transcribe CCR2 but fail to translate it into protein. Similar results were seen in NK cells and T cells (data not shown). Additional signals might initiate CCR2 translation in these monocytes and thus provide the potential to rapidly upregulate CCR2 expression.

To investigate the functions of monocyte subsets, we crossed the CCR2-RFP mice with CX3CR1-GFP knock-in mice [23] to create *Ccr2^RFP/+^Cx3cr1^GFP/+^* mice. These “two-color” mice were examined in three pathological settings: *Apoe* deficiency on a high-fat diet, thioglycollate induced-peritonitis (localized sterile acute inflammation), and EAE (localized organ-specific autoimmune inflammation). Overall, the findings support the notion that CCR2 and CX3CR1 define functionally distinct monocyte subsets and for the first time we provide a model to visualize monocytes subsets in situ within brain infiltrates.

Under the steady-state, low-level inflammatory conditions of a high-fat diet, Ly6C^hi^ monocytes had higher RFP intensity and lower GFP intensity than Ly6C^lo^ cells, allowing the subsets to be clearly distinguished by their RFP/GFP profiles. To investigate the trafficking of monocyte subsets in an acute inflammatory model, we used thioglycollate to induce sterile peritonitis. At 24 h, the vast majority of monocyte/macrophages recruited to the peritoneum in *Ccr2^+/RFP^*Cx3cr1*^+/GFP^* mice expressed RFP and GFP. Because CCR2 is rapidly downregulated when monocytes differentiate into macrophages [33], these results suggest that the RFP^hi^ cells were newly arrived monocyte/macrophages and show that the RFP and GFP markers are useful for tracking migrating monocytes. Interestingly, the absence of CX3CR1 in the *Ccr2^RFP/+^*Cx3cr1*^GFP/GFP^* mice had little or no effect on the RFP/GFP profile or the number of recruited cells. By contrast, deletion of CCR2 in *Ccr2^RFP/RFP^Cx3cr1^+/GFP^* mice drastically reduced peritoneal macrophage recruitment.

CCR2 and CX3CR1 have been implicated as disease modifiers in EAE, a model for the inflammatory aspects of multiple sclerosis. EAE severity is restricted in CCR2-deficient mice on a mixed C57Bl/6 and 129/J background and in CCL2-deficient (MCP-1/JE) mice [34]–[36]. It has been proposed that CCR2^+^ monocytes that enter the CNS during EAE differentiate into macrophages and DCs, thereby promoting EAE pathogenesis [17], [37]. In contrast, CX3CR1 deficiency exacerbates EAE because regulatory NK cells are not recruited to the inflamed CNS [26].

Our EAE experiments revealed physiologically relevant and mechanistically intriguing functions of CCR2 expression and regulation of CNS myeloid inflammation. We predicted that CCR2 deficiency would preferentially reduce Ly6C^hi^ monocyte infiltration into the CNS of mice with EAE. To test this hypothesis, we compared the pathogenesis of EAE in *Ccr2^RFP/+^Cx3cr1^GFP/+^* and CCR2-deficient *Ccr2^RFP/RFP^Cx3cr1^GFP/+^* mice. Mice deficient in both CCR2 and CX3CR1 (*Ccr2^RFP/RFP^Cx3cr1^GFP/GFP^ mice*) were included to probe the interactions between these receptors in EAE. As predicted, Ly6C^hi^ monocytes were virtually absent from the CNS of the *Ccr2^RFP/RFP^Cx3cr1^GFP/+^* mice with EAE. Unexpectedly, the total numbers of leukocytes infiltrating the CNS were indistinguishable in control and CCR2-deficient mice. Detailed analysis of CNS infiltrates by flow cytometry and fluorescence microscopy revealed unanticipated characteristics of myeloid cell populations in the CNS.

First, by evaluating monocyte/macrophage/microglial chemokine receptor expression in EAE infiltrates, we found that CD45^lo^ microglia invariably expressed CX3CR1 but not CCR2 in both healthy and diseased CNS tissues. Thus, CCR2-RFP and CX3CR1-GFP can differentiate resident microglia (GFP only) from infiltrated monocyte-derived cells (RFP^+^ with various degrees of GFP expression). Resident microglia and infiltrating monocyte-derived macrophages fulfill very different functions, as most pointedly indicated by analysis of their functions in Alzheimer's disease models [38]. Therefore, the capacity to distinguish resident microglia from infiltrating monocytes will be valuable for functional characterizations.

Second, neutrophils, not Ly6C^lo^ monocytes, replaced Ly6C^hi^ monocytes in the brains of *Ccr2^RFP/RFP^Cx3cr1^GFP/+^* mice with EAE, accounting for the equivalent number of total leukocytes. Thus, our findings reveal that Ly6C^lo^/CX3CR1^+^ monocytes are relatively excluded from the inflamed CNS of mice with EAE, regardless of CCR2 expression. Therefore, Ly6C^hi^ and Ly6C^lo^ monocytes do no appear to contribute sequentially to tissue inflammation or tissue remodeling/repair in the CNS as reported in cardiac ischemic injury (16). We propose that endogenous microglia perform the repair and remodeling functions carried out by Ly6C^lo^ monocytes in peripheral organs. Thus, CCR2 antagonists [39] will likely affect CCR2^+^ infiltrating monocytes but not resident CX3CR1^+^ microglia.

A third major finding, made possible by the use of RFP as a surrogate marker for CCR2, was that the absence of CCR2 prevents monocytes from leaving the vasculature and entering the perivascular space. There is considerable controversy regarding whether CCR2 mediates monocyte trafficking out of hematopoietic organs into the blood, trafficking from blood to inflamed organs, or both [12], [13]. CCR2 ligands, such as MCP-1, are made by parenchymal cells of the CNS [40], consistent with the hypothesis that the CCL2/CCR2 axis is critical for monocyte recruitment to the inflamed CNS from the blood [41].

Finally, RFP and GFP expression in infiltrated cells in the CNS overlapped with the patterns observed in the blood. In particular, mononuclear cells in the perivascular region of the brain displayed a range of RFP/GFP ratios consistent with those in the blood (Figures 2 5, and 6). For example, in FACS analyses, Ly6C^hi^ cells in the diseased CNS of *Ccr2^RFP/+^Cx3cr1^GFP/+^* mice with EAE were RFP^hi^ and GFP^med/lo^, while the infrequent Ly6C^lo^ cells were GFP^hi^/RFP^lo^ (Figure 2I). We used 7/4 as a surrogate for Ly6C staining in brain sections and found a corresponding correlation between the presence of RFP and GFP depending on the 7/4 status of the monocyte (Figure 6).

In *Ccr2^RFP/RFP^Cx3cr1^GFP/+^* mice, the onset of EAE occurred 5 days later than in *Ccr2^RFP/+^Cx3cr1^GFP/+^* control mice, but reached similar peak severity with the same kinetics as in littermate controls. The delay in EAE development was presumably due to the absence of CCR2^+^ monocytes in the CNS. Their particular contribution is a subject of future work, but it has been suggested that they express inflammatory mediators and can convert to DCs [17], [37]. In support of this hypothesis, CCR2-deficient mice had 50% fewer CD11c+ cells in the brain with EAE, as compared with CCR2+ mice at the same sate of disease (unpublished data).

Spinal cord total inflammatory burden was not affected by CCR2 deficiency (Figure S2). This finding most likely accounts for the similar peak disease severity in the *Ccr2^+/RFP^* and *Ccr2^RFP/RFP^* mice, since the neurological signs of EAE arise from spinal-cord involvement [30]. These results generally correspond to those reported in CCL2 knockout mice [35]. However, in the current study, we analyzed fully-back-crossed knockout mice at 24 weeks of age, which may explain the differences in neurobehavioral results between our studies and some previous studies [34], [36], [42]. In double-receptor knockout mice (*Ccr2^RFP/RFP^Cx3cr1^GFP/GFP^*), neurological signs of EAE were indistinguishable from those of CCR2-deficient *Ccr2^RFP/RFP^Cx3cr1^+/GFP^* mice, indicating that CCR2 plays a dominant role in EAE severity (data not shown).

There are several limitations to the usefulness of this model for *in vivo* imaging. First, because some T cells and NK cells express CCR2 transcripts and are thus RFP-positive, it is not possible to conclude that all red cells in an image are monocytes. Depending on the specific setting, additional markers may have to be included to allow more precise identification of the cells, as illustrated in the EAE experiments in Figure 6, in which we found that the vast majority, but not all RFP^+^ cells were also 7/4^+^. In addition, although RFP intensity correlates well with Ly6C intensity, the overlap is not perfect, and some RFP^bright^ cells may be Ly6^lo^. Nonetheless, the ability to simultaneously image CCR2 and CX3CR1 expression in monocytes and other leukocytes represents a powerful new tool for investigating complex pathophysiology.

In summary, our findings confirm, refine, and extend the concept of monocyte heterogeneity. We created and characterized a transgenic mouse model that allows simultaneous tracking of two key chemokine receptors, CCR2 and CX3CR1 and enumeration of distinct monocyte and myeloid subsets based on CCR2-RFP/CX3CR1-GFP expression. We showed that CCR2 expression is controlled at a post-transcriptional stage in some Ly6C^lo^ monocytes and NK and T cells. We found that Ly6C^hi^ monocytes fail to enter the CNS of CCR2deficient mice during EAE and are mainly replaced by neutrophils, not Ly6C^lo^ monocytes. We hypothesize that CCR2^+^ Ly6C^hi^ monocytes initiate and maintain neuroinflammatory responses, while tissue remodeling is mediated by CX3CR1^+^ microglia. The CCR2-RFP/CX3CR1-GFP reporter mice should be useful for studying the roles of CCR2 and CX3CR1 in myeloid cell recruitment in a wide range of inflammatory diseases. The expression of CCR2 transcripts in some NK and T cells is of interest and must be taken into account when using this model.

## Materials and Methods

### Ethics Statement

All animals were handled in strict accordance with good animal practice as defined by the relevant national and/or local animal welfare bodies, and all animal work was approved by the appropriate committees: IACUC University of California, San Francisco APPROVAL NUMBER: AN079763-02; IACUC Cleveland Clinic APPROVAL NUMBER: ARC 07541; IACUC University of Texas, San Antonio APPROVAL NUMBER: MU060-08/12A0.

### Mice

CCR2^RFP^ mice were created as described [25] (see Figure 1 and Text S1). Founder mice were crossed with Cre-deleter mice [43] to remove the *neo* gene and backcrossed onto the C57Bl/6 line nine times. To generate homozygous *Ccr2^RFP/RFP^Cx3cr1^GFP/GFP^* mice, we crossed *Ccr2^RFP/RFP^* with *Cx3cr1^GFP/GFP^* C57Bl/6 mice (a gift of D.R. Littman), and the progeny were backcrossed onto C57Bl/6. Mice that had undergone chromosome recombination between the CCR2 and CX3CR1 loci were selected by being positive for both RFP and GFP by flow cytometry of tail vein blood.

Unless stated otherwise, all mice were backcrossed seven to nine times on C57Bl/6 and were 2–6 months of age at sacrifice. Some mice were crossed with C57Bl/6 *Apo*e^−/−^ mice. These mice were fed a Western diet (42% of calories from fat) (Harlan Teklad, TD88137) for 8 weeks, starting at 6–8 weeks of age. All other mice were fed standard chow. Mice were bred at the Gladstone Institutes and the Biological Resources Unit, Cleveland Clinic, Lerner Research Institute. Animal experiments were performed according to the protocols approved by the Institutional Animal Care and Use Committee at the Cleveland Clinic, UCSF, and UTSA following the National Institute of Health guidelines for animal care. Mice were genotyped by PCR using tail DNA, and chemokine receptor–specific primers (Invitrogen, Carlsbad,CA) (Table S1).

### EAE induction

At 12–24 weeks of age, EAE was induced in male and female mice weighing less than 32 g as described [26] and monitored for signs of disease as described [35]: 0, no disease; 1, decreased tail tone and/or poor righting ability; 2, abnormal gait and/or hind limb weakness; 2.5, partial hind limb paralysis; 3, complete hind limb paralysis; 3.5, ascending paralysis; 4, tetraplegia; 5, death. Mice were sacrificed at a score of 3–4.

### Microscopy

Brains and spinal cords were either perfused with phosphate-buffered saline (PBS) and frozen whole in OCT or perfused with or 4% PFA and processed into free-floating 30-µm sections, as described [26]. Sections were stained for CD45, as described [44]. For details on histological methods, please see Text S1. To visualize the distribution of cells expressing GFP (CX3CR1) and RFP (CCR2), free-floating sections were stained with 15 nM DAPI for 5 min, and whole-brain sections were scanned using a Leica DM5500B with motorized x,y stage. High-power confocal images are shown as projections of 7–10-µm z stacks at 40x magnification and scanned at a step size of 1–1.5 µm. In some experiments, brain sections were incubated overnight at 4°C with 7–4 antibody (Cedarlane, Ontario, Canada), washed, and incubated at room temperature for 2 h with biotinylated-rabbit anti rat secondary antibody (Jackson ImmunoResearch). After additional washes, tissues were incubated at room temperature for 1 h with strepatvidin-Cy5 conjugated, rinsed and mounted in Fluorsave reagent (Calbiochem). Tissues were imaged at 40x magnification on a Zeiss 510 confocal microscope.

### Flow cytometry

For flow cytometric analysis of CNS tissues, mice were perfused with cold Hank's balanced salt solution. The brains were dissected and the spinal cords were flushed with 0.5 ml of cold PBS. Tissues were homogenized with Dounce homogenizers, and cells were separated in a discontinuous 30/70 Percoll gradient [45]. Cells were collected from the 30/70 interphase and washed in PBS.

Blood was collected by cardiac puncture of anesthetized mice^,^ depleted of red blood cells in lysis buffer (0.15 mol/l NH4Cl, 1 mmol/l KHCO3, 0.1 mmol/l EDTA), and washed in staining buffer (PBS/0.1% BSA). For flow details, please see Text S1.

### Sterile peritonitis model

Peritonitis was induced with thioglycollate as described [25]. Peritoneal leukocytes were collected 24 h later by flushing the peritoneal cavity with 10 ml of PBS. Peritoneal cells were washed with staining buffer and stained for F4/80 and 7-4 as described above.

### Quantitative RT-PCR

RNA was isolated from FACS-sorted cell populations with the Arcturus PicoPure kit (Molecular Devices, Sunnyvale, CA) according to the manufacturer's instructions. Quantity was assessed with a Nanodrop 1000, and RNA was reverse transcribed with TaqMan reverse-transcription reagents. Quantitative real-time PCR was carried out with TaqMan PCR Master Mix, TaqMan gene expression assays, and an Applied Biosystems 7900HT thermal cycler. All TaqMan reagents were from Applied Biosystems. Reactions were run in quadruplicate, and CCR2 and RFP mRNA expression levels were normalized to beta-actin.

### Statistical analysis

Data are presented as mean ± SEM. Differences between *Cx3cr1^+/−^Ccr2^+/−^* and *Cx3cr1^+/−^Ccr2^−/−^* mice were analyzed with a two-tailed nonparametric Mann-Whitney test.

## Supporting Information

Text S1 Supporting Materials and Methods.
(0.03 MB DOC)

Table S1 Primers used for tail-DNA genotyping. *Primers A-B amplify the wild type allele, whereas primers B-C identify the knockout allele.
(0.03 MB DOC)

Figure S1 Analyses of cytoplasmic CCR2. WT, Ccr2RFP/+, and Ccr2RFP/RFP mice were bred onto the Apoe−/− background and fed a high-fat diet for 8 weeks. Intact and permeabilized monocytes, identified by CD115 staining, were stained for CCR2 and Ly6C. Boxes indicate demarcation of monocytes into Ly6Chi and Ly6Clo populations. Dashed line indicates the cutoff for positive CCR2 staining, using Ccr2RFP/RFP cells as the negative control. Permeabilization did not increase background staining in the CCR2 knockout mice (not shown). Results are representative of four experiments.
(0.50 MB EPS)

Figure S2 Analysis of spinal inflammation in CCR2-deficient mice. Spinal cord tissues were fixed and stained with anti-CD45 antibodies to compare the distribution of inflammatory cells in double heterozygous Ccr2RFP/+ Cx3cr1GFP/+ mice (A,B) and Ccr2RFP/RFP Cx3cr1GFP/+ mice (C,D) at peak EAE disease. The extent of disease was similar in both genotypes. WM: white matter. GM: gray matter.
(1.97 MB EPS)

## References

[1] Auffray C, Fogg D, Garfa M, Elain G, Join-Lambert O (2007). Monitoring of blood vessels and tissues by a population of monocytes with patrolling behavior.. Science.

[2] Geissmann F, Jung S, Littman DR (2003). Blood monocytes consist of two principal subsets with distinct migratory properties.. Immunity.

[3] Qu C, Edwards E, Tacke F, Angeli V, Llodrá J (2004). Role of CCR8 and other chemokine pathways in the migration of monocyte-derived dendritic cells to lymph nodes.. J Exp Med.

[4] Randolph GJ, Inaba K, Robbiani DF, Steinman RM, Muller WA (1999). Differentiation of phagocytic monocytes into lymph node dendritic cells in vivo.. Immunity.

[5] Swirski FK, Libby P, Aikawa E, Alcaide P, Luscinskas FW (2007). Ly-6Chi monocytes dominate hypercholesterolemia-associated monocytosis and give rise to macrophages in atheromata.. J Clin Invest.

[6] Banchereau J, Steinman R (1998). Dendritic cells and the control of immunity.. Nature.

[7] Gordon S (2002). Pattern recognition receptors: doubling up for the innate immune response.. Cell.

[8] Sunderkötter C, Nikolic T, Dillon MJ, van Rooijen N, Stehling M (2004). Subpopulations of mouse blood monocytes differ in maturation stage and inflammatory response.. J Immunol.

[9] Grage-Griebenow E, Flad H, Ernst M (2001). Heterogeneity of human peripheral blood monocyte subsets.. J Leukoc Biol.

[10] Grage-Griebenow E, Flad H, Ernst M, Bzowska M, Skrzeczyñska J (2000). Human MO subsets as defined by expression of CD64 and CD16 differ in phagocytic activity and generation of oxygen intermediates.. Immunobiology.

[11] Ziegler-Heitbrock H (2000). Definition of human blood monocytes.. J Leukoc Biol.

[12] Serbina N, Pamer E (2006). Monocyte emigration from bone marrow during bacterial infection requires signals mediated by chemokine receptor CCR2.. Nat Immunol.

[13] Tsou CL, Peters W, Si Y, Slaymaker S, Aslanian AM (2007). Critical roles for CCR2 and MCP-3 in monocyte mobilization from bone marrow and recruitment to inflammatory sites.. J Clin Invest.

[14] Weber C, Belge K, von Hundelshausen P, Draude G, Steppich B (2000). Differential chemokine receptor expression and function in human monocyte subpopulations.. J Leukoc Biol.

[15] Ancuta P, Rao R, Moses A, Mehle A, Shaw SK (2003). Fractalkine preferentially mediates arrest and migration of CD16^+^ monocytes.. J Exp Med.

[16] Nahrendorf M, Swirski F, Aikawa E, Stangenberg L, Wurdinger T (2007). The healing myocardium sequentially mobilizes two monocyte subsets with divergent and complementary functions.. J Exp Med.

[17] Mildner A, Mack M, Schmidt H, Brück W, Djukic M (2009). CCR2+Ly-6Chi monocytes are crucial for the effector phase of autoimmunity in the central nervous system.. Brain.

[18] Tacke F, Alvarez D, Kaplan TJ, Jakubzick C, Spanbroek R (2007). Monocyte subsets differentially employ CCR2, CCR5, and CX3CR1 to accumulate within atherosclerotic plaques.. J Clin Invest.

[19] Auffray C, Sieweke M, Geissmann F (2009). Blood monocytes: development, heterogeneity, and relationship with dendritic cells.. Annu Rev Immunol.

[20] Gordon S, Taylor PR (2005). Monocyte and macrophage heterogeneity.. Nat Rev Immunol.

[21] Varol C, Yona S, Jung S (2009). Origins and tissue-context-dependent fates of blood monocytes.. Immunol Cell Biol.

[22] Tacke F, Randolph GJ (2006). Migratory fate and differentiation of blood monocyte subsets.. Immunobiology.

[23] Jung S, Aliberti J, Graemmel P, Sunshine MJ, Kreutzberg GW (2000). Analysis of fractalkine receptor CX_3_CR1 function by targeted deletion and green fluorescent protein reporter gene insertion.. Mol Cell Biol.

[24] Campbell R, Tour O, Palmer A, Steinbach P, Baird G (2002). A monomeric red fluorescent protein.. Proc Natl Acad Sci U S A.

[25] Boring L, Gosling J, Chensue SW, Kunkel SL, Farese RV (1997). Impaired monocyte migration and reduced type 1 (Th1) cytokine responses in C-C chemokine receptor 2 knockout mice.. J Clin Invest.

[26] Huang D, Shi F, Jung S, Pien G, Wang J (2006). The neuronal chemokine CX3CL1/fractalkineselectively recruits NK cells that modify experimental autoimmune encephalomyelitis within the central nervous system.. FASEB J.

[27] Glabinski A, Tani M, Strieter R, Tuohy V, Ransohoff R (1997). Synchronous synthesis of alpha-and beta-chemokines by cells of diverse lineage in the central nervous system of mice with relapses of chronic experimental autoimmune encephalomyelitis.. Am J Pathol.

[28] Cardona A, Pioro E, Sasse M, Kostenko V, Cardona S (2006). Control of microglial neurotoxicity by the fractalkine receptor.. Nat Neurosci.

[29] Sedgwick J, Schwender S, Imrich H, Dörries R, Butcher G (1991). Isolation and direct characterization of resident microglial cells from the normal and inflamed central nervous system.. Proc Natl Acad Sci U S A.

[30] Glabinski A, Tani M, Tuohy V, Ransohoff R (1997). Murine experimental autoimmune encephalomyelitis: a model of immune-mediated inflammation and multiple sclerosis.. Methods Enzymol.

[31] Rostène W, Kitabgi P, Parsadaniantz S (2007). Chemokines: a new class of neuromodulator.. Nat Rev Neurosci.

[32] Jung H, Bhangoo S, Banisadr G, Freitag C, Ren D (2009). Visualization of chemokine receptor activation in transgenic mice reveals peripheral activation of CCR2 receptors in states of neuropathic pain.. J Neurosci.

[33] Wong L-M, Myers SJ, Tsou C-L, Gosling J, Arai H (1997). Organization and differential expression of the human monocyte chemoattractant protein 1 receptor gene. Evidence for the role of the carboxyl-terminal tail in receptor trafficking.. J Biol Chem.

[34] Fife B, Huffnagle G, Kuziel W, Karpus W (2000). CC chemokine receptor 2 is critical for induction of experimental autoimmune encephalomyelitis.. J Exp Med.

[35] Huang DR, Wang J, Kivisakk P, Rollins BJ, Ransohoff RM (2001). Absenceof monocyte chemoattractant protein 1 in mice leads to decreased local macrophage recruitment and antigen-specific T helper cell type 1 immune response in experimental autoimmune encephalomyelitis.. J Exp Med.

[36] Izikson L, Klein RS, Charo IF, Weiner HL, Luster AD (2000). Resistance to experimental autoimmune encephalomyelitis in mice lacking the CC chemokine receptor (CCR)2.. J Exp Med.

[37] King I, Dickendesher T, Segal B (2009). Circulating Ly-6C+ myeloid precursors migrate to the CNS and play a pathogenic role during autoimmune demyelinating disease.. Blood.

[38] El Khoury J, Toft M, Hickman S, Means T, Terada K (2007). Ccr2 deficiency impairs microglial accumulation and accelerates progression of Alzheimer-like disease.. Nat Med.

[39] Charo IF, Ransohoff RM (2006). The many roles of chemokines and chemokine receptors in inflammation.. N Engl J Med.

[40] Ransohoff R, Hamilton T, Tani M, Stoler M, Shick H (1993). Astrocyte expression of mRNA encoding cytokines IP10 and JE/MCP-1 in experimental autoimmune encephalomyelitis.. FASEB J.

[41] Mahad DJ, Ransohoff RM (2003). The role of MCP-1 (CCL2) and CCR2 in multiple sclerosis and experimental autoimmune encephalomyelitis (EAE).. Semin Immunol.

[42] Gaupp S, Pitt D, Kuziel W, Cannella B, Raine C (2003). Experimental autoimmune encephalomyelitis (EAE) in CCR2(-/-) mice: susceptibility in multiple strains.. Am J Pathol.

[43] Lakso M, Pichel JG, Gorman JR, Sauer B, Okamoto Y (1996). Efficient *in vivo* manipulation of mouse genomic sequences at the zygote stage.. Proc Natl Acad Sci U S A.

[44] Cardona A, Restrepo B, Jaramillo J, Teale J (1999). Development of an animal model for neurocysticercosis: immune response in the central nervous system is characterized by a predominance of gamma delta T cells.. J Immunol.

[45] Cardona A, Huang D, Sasse M, Ransohoff R (2006). Isolation of murinemicroglial cells for RNA analysis or flow cytometry.. Nat Protoc.

