# Correction: Selective Chemokine Receptor Usage by Central Nervous System Myeloid Cells in CCR2-Red Fluorescent Protein Knock-In Mice

**DOI:** 10.1371/journal.pone.0176931

**Published:** 2017-04-27

**Authors:** Noah Saederup, Astrid E. Cardona, Kelsey Croft, Makiko Mizutani, Anne C. Cotleur, Chia-Lin Tsou, Richard M. Ransohoff, Israel F. Charo

The Supporting Information file S1 Text is incorrectly duplicated as S1 Table. Please see the correct S1 Table here.

## Supporting information

S1 Table Primers used for tail-DNA genotyping. *Primers A-B amplify the wild type allele, whereas primers B-C identify the knockout allele.
(PDF)
