# Supplementary material for: Selective Chemokine Receptor Usage by Central Nervous System Myeloid Cells in CCR2-Red Fluorescent Protein Knock-In Mice
Source: PLoS One. 2010 Oct 27;5(10):e13693. doi: 10.1371/journal.pone.0013693 (PMC2965160; doi:10.1371/journal.pone.0013693)
Supplement: Text S1 — Supporting Materials and Methods. (0.03 MB DOC) [file pone.0013693.s001.doc]

**Text S1**

## Mice

The CCR2^RFP^ targeting vector contains a 4.351-kb PCR-amplified (Expand High Fidelity Kit; Roche Applied Science) genomic segment immediately upstream of the CCR2 start codon. This amplicon was connected by overlapping PCR amplification to a 675-bp monomeric RFP expression cassette with a 3’ SV40 polyA signal (generous gift of Dr. R. Tsien). This amplicon was ligated upstream of the neomycin-resistance gene (flanked by *loxP* sites) in vector pSV-Neo-TK. A 3.319-kb PCR-generated genomic segment containing the 3’ end of the CCR2 open reading frame, starting from nucleotide position 280, was inserted downstream of the mRFP/Neo cassette. Removal of the *neo* gene by breeding with *Cre*-deleter mice was confirmed by PCR of the region spanning the *neo* gene and the flanking CCR2 exon.

Mice were genotyped by Southern blot using *Pst*I-digested tail DNA and a probe generated by PCR spanning the genomic region of CCR2 corresponding to positions bp 184633 to 185394 (Acc# NT_039483). For colony maintenance, mice were genotyped by PCR using tail DNA, and chemokine receptor–specific primers (Invitrogen, Carlsbad, CA) (Table S1). PCR reactions were prepared in a volume of 10 μl with the AmpliTaq-Gold system (Applied Biosystems, Foster City, CA) and resolved in 1% agarose gels.

**Flow Cytometry**

CNS cell pellets were resuspended in cell-staining buffer (Biologend, San Diego, CA), incubated on ice for 5 min with anti-mouse CD16/CD32 (Clone 2.4G2, BD Pharmingen, Franklin Lakes, NJ) to block Fc receptors, and then incubated on ice for 30 min with a mix of fluorochrome-conjugated anti-mouse antibodies: CD45-PerCP (1:100; Clone 30-F11, BD Pharmingen), CD115-PE (1:10; Clone AFS98, eBioscience), CD11c-PeCy7 (1:50; Clone N418, eBioscience), and Ly6C-Alexa 647 (1:50; Clone ER-MP20, AbD Serotec). After washes, cells were resuspended in 2% paraformaldehyde and analyzed in a LSR-II (BD Biosciences, Franklin Lakes, NJ) configured with an argon 488 laser with a 505 LP dichroic and 525/50 filter to detect GFP and a 670 LP dichroic with a 695/40 filter for PerCP; HeNe 639 laser with a 755 LP and 780/60 filter to detect APC-Cy7, and 660/20 filter to detect APC and Alexa 647; and a green 532 laser with a 735 LP dichroic and a 750-810 filter to detect Pe-Cy7 and 575/26 to detect PE.

Leukocytes were incubated with rat anti-mouse CD16/CD32 (1:500; BD Biosciences, San Jose, CA) or goat anti-mouse CD16/32 (1:250; R&D Systems, Minneapolis, MN) for 15 min to block Fc receptors. Leukocytes were incubated for 30 min at 4°C with combinations of different fluorescently labeled antibodies, including GR-1-PeCy7 (1:500), Ly6C-FITC (1:100), CD3-PE (1:100), NK1.1-APC (1:100), biotinylated goat anti-rat IgG (all from BD Biosciences), F4/80-APC (1:100, or 2 µg/ml; Caltag Laboratories, South San Francisco, CA), F4/80-PE (1:100; Caltag Laboratories), CD115-PE/CD115-APC (1:200, eBioscience; San Diego, CA), anti-CCR2 (Clone MC-21, a gift of M. Mack) and streptavidin-APC/PE (1:500/1:10000, BD Biosciences). Cells were washed three times between staining steps. Leukocytes were sorted on a FACSAria2 configured with three lasers (488 nm, 630 nm, and 532 nm), using NK1.1 for NK cells, CD3 for T cells, and CD115 and CCR2 for monocytes. Samples were analyzed with FlowJo software (Tree Start, Ashland, OR).

## Microscopy

Mice were perfused with 10% sucrose followed by 50 ml of 4% paraformaldehyde. Dissected brains and spinal cords were postfixed overnight and cryoprotected in 20% glycerol in 80 mM phosphate buffer, pH 7.6, for 48 h at 4˚C. Free-floating 30-µm sections were prepared in a freezing microtome and stored at –20˚C until use. Tissues were stained overnight at 4˚C with anti-mouse CD45 antibodies (clone IBL-3/16, AbD Serotec, Raleigh, NC), incubated with biotin-labeled secondary antibody, and developed with avidin-biotin peroxidase system (Vector Laboratories, Burlingame, CA) with DAB as a substrate (Invitrogen, Carlsbad, CA). Sections were imaged with a Leica microscope.
