# Supplementary material for: Correction: Selective Chemokine Receptor Usage by Central Nervous System Myeloid Cells in CCR2-Red Fluorescent Protein Knock-In Mice
Source: PLoS One. 2017 Apr 27;12(4):e0176931. doi: 10.1371/journal.pone.0176931 (PMC5407805; doi:10.1371/journal.pone.0176931)
Supplement: S1 Table — *Primers A-B amplify the wild type allele, whereas primers B-C identify the knockout allele. (PDF) [file pone.0176931.s001.pdf]

Supplementary Table1. Primers used for tail-DNA genotyping

Line	Primers*
CX3CR1	(A) TTC ACG TTC GGT CTG GTG GG
	(B) GGT TCC TAG TGG AGC TAG GG
	(C) GAT CAC TCT CGG CAT GGA CG
CCR2	(A) CCT TCA TCA AGC TCT TGG
	(B) GTG TGT GCA GGT TCC AAT GGA G
	(C) GGA AGA CAA TAG CAG GCA TGC

*Primers A-B amplify the wild type allele, whereas primers B-C identify the knockout allele.
